# MIntO: A Modular and Scalable Pipeline For Microbiome Metagenomic and Metatranscriptomic Data Integration

**DOI:** 10.3389/fbinf.2022.846922

**Published:** 2022-05-10

**Authors:** Carmen Saenz, Eleonora Nigro, Vithiagaran Gunalan, Manimozhiyan Arumugam

**Affiliations:** Novo Nordisk Foundation Center for Basic Metabolic Research, Faculty of Health and Medical Sciences, University of Copenhagen, Copenhagen, Denmark

**Keywords:** omics integration, metagenomic, metatranscriptomic, pipeline, gene expression, community turnover, microbial ecology, microbiome

## Abstract

Omics technologies have revolutionized microbiome research allowing the characterization of complex microbial communities in different biomes without requiring their cultivation. As a consequence, there has been a great increase in the generation of omics data from metagenomes and metatranscriptomes. However, pre-processing and analysis of these data have been limited by the availability of computational resources, bioinformatics expertise and standardized computational workflows to obtain consistent results that are comparable across different studies. Here, we introduce MIntO (Microbiome Integrated meta-Omics), a highly versatile pipeline that integrates metagenomic and metatranscriptomic data in a scalable way. The distinctive feature of this pipeline is the computation of gene expression profile through integrating metagenomic and metatranscriptomic data taking into account the community turnover and gene expression variations to disentangle the mechanisms that shape the metatranscriptome across time and between conditions. The modular design of MIntO enables users to run the pipeline using three available modes based on the input data and the experimental design, including *de novo* assembly leading to metagenome-assembled genomes. The integrated pipeline will be relevant to provide unique biochemical insights into microbial ecology by linking functions to retrieved genomes and to examine gene expression variation. Functional characterization of community members will be crucial to increase our knowledge of the microbiome’s contribution to human health and environment. MIntO v1.0.1 is available at https://github.com/arumugamlab/MIntO.

## Introduction

The human microbiome is a complex congregation of microbes comprising trillions of microbial cells present in our bodies (Bashan et al., 2016). Microbe-microbe and microbe-host interactions confer a variety of physiological benefits to the hosts and impact their susceptibility to disease. For instance, the microbial niche can provide metabolic functions different from the host genome, most of which are encoded by genes that have not yet been discovered (Nicholson et al., 2012; Donia and Fischbach, 2015).

Studying these microbial communities is a challenging task, which has recently been made easier by high-throughput sequencing approaches which generate omics data such as metagenomes and metatranscriptomes. These omics methods have revolutionized microbiome research by allowing the characterization of complex microbial communities in different biomes without requiring their cultivation. Metagenomic data enables the genomic and taxonomic characterization of microbial community composition and, depending on the sequencing strategy employed, can allow the recovery of Metagenome-Assembled Genomes (MAGs) (Almeida et al., 2019; Stewart et al., 2019; Saheb Kashaf et al., 2022). However, it can only unravel the functional potential in a sample (Quince et al., 2017). In contrast, metatranscriptomic data identifies the pool of genes that are transcribed under a specific condition, which gives a more accurate picture of the processes and molecular activity occurring in the microbial community (Satinsky et al., 2014; Salazar et al., 2019). Hence, by analyzing both metagenomes and metatranscriptomes, we can have deeper insights into the functional potential as well as the actual activity of microbial communities (Wang et al., 2020; Tláskal et al., 2021).

In recent years, the application of high-throughput sequencing approaches in microbiome research has greatly increased together with the generation of large amounts of data (Qin et al., 2010; Human Microbiome Project Consortium, 2012; Pasolli et al., 2019). As a consequence, the pre-processing and analysis of such data have been limited by the availability of computational resources and bioinformatics expertise. In addition, there is a lack of standardized protocols to handle and analyze multi-omics data sets in a more consistent manner, making the comparisons between different studies and findings more challenging. Standardizing the way omics data are handled ensures a degree of consistency of the results across different studies. Furthermore, making the workflows semi-automatic will allow the analysis of complex microbial communities by users with limited bioinformatic skills.

Standard metagenomic and metatranscriptomic approaches entail 1) read curation, 2) *de novo* assembly and/or co-assembly, 3) binning, 4) gene prediction, 5) annotation of predicted genes at taxonomic and functional level and 6) quantification of gene abundances and transcripts. However, most of the computational pipelines developed so far can only analyze metagenomic or metatranscriptomic data individually and only few, reported in Table 1, can handle both meta-omics data (Kim et al., 2016; Narayanasamy et al., 2016; Tamames and Puente-Sánchez, 2018; Salazar et al., 2019; Sequeira et al., 2019; Van Damme et al., 2021). Furthermore, only one of them (Van Damme et al., 2021) can combine two sequencing technologies (Nanopore or long-sequences and Illumina or short-sequences) to recover MAGs.

**TABLE 1:** Features of pipelines that handle metagenomic and metatranscriptomic data in comparison to MIntO: Steps, capacities and approaches.

	FMAP Kim et al. (2016); Salazar et al. (2019)	IMP Narayanasamy et al. (2016)	MOSCA Sequeira et al. (2019)	SqueezeMeta Tamames and Puente-Sánchez, (2018)	MUFFIN Van Damme et al. (2021)	MIntO (2021)
data source	short reads	paired-end short reads	paired-end short reads	paired-end short reads	paired-end Illumina reads (short reads) and Nanopore-based reads (long reads)	paired-end Illumina reads (short reads) and Nanopore-based reads (long reads)
quality and read length control	only quality control	Yes	Yes	Yes	Yes	Yes
host genome removal	only human genome removal	Yes	No	No	No	Yes
rRNA removal	No	Yes	Yes	No	No	Yes
taxonomy assignment	No	Yes	Yes	Yes	Yes	Yes
*de novo* assembly/co-assembly	No	Yes	Yes	Yes	combining short and long reads	optionally, include long reads
binning	No	Yes	Yes	Yes	Yes	Yes
gene prediction	Yes	Yes	Yes	Yes	Yes	Yes
function annotation	Yes	Yes	Yes	Yes	Yes	Yes
alignment to reference database/genomes	alignment to reference database	Yes	No	No	No	Yes
alignment to retrieved MAGs	No	Yes	Yes	Yes	Yes	Yes
normalization	RPKM	RPKM	TMM, RLE	RPKM	TPM	TPM, Marker genes
visualization	Yes	Yes	Yes	No	Yes	Yes
local installation	Yes	Yes	Yes	Yes	Yes	Yes
gene expression computation	No	No	No	No	No	Yes
differential analysis/Downstream analysis	differentially-abundant genes analysis	No	differential gene expression analysis	No	No	No
Software dependencies installed by the user before using the pipeline	Perl, R, Statistics::R, DIAMOND or USEARCH, Bio::DB::Taxonomy, XML::LibXML	Python3, pip, impy, Conda, Docker/Singularity	MOSGUITO, and Conda, or Docker/Singularity	Conda	Nextflow and Conda or Docker/Singularity	FetchMGs, Conda

Overall, the pipelines shown in Table 1 integrate metagenomic and metatranscriptomic data by comparing the abundances of genes and their respective transcripts. To the best of our knowledge, none of these (Table 1) considers the community composition and gene expression alterations as the underlying processes that shape the community transcript levels (Salazar et al., 2019) when integrating metagenomic and metatranscriptomic data. However, perturbations of the transcript levels can be a consequence of two factors: the variation in the expression of genes encoded by the organisms in the community, and/or by changes in the abundance of these members and their related genes in a process known as community turnover (Satinsky et al., 2014; Salazar et al., 2019). Hence, the integration of abundances of genes and the respective transcripts represents the gene expression profiles, which are the relative amount of transcripts per gene in a specific time (Salazar et al., 2019). Additionally, being able to recover genomes from metagenomic raw reads is crucial for an optimal computation of gene expression levels and provides a more accurate ecological description of the community’s functioning (Tamames and Puente-Sánchez, 2018).

**FIGURE 1:**
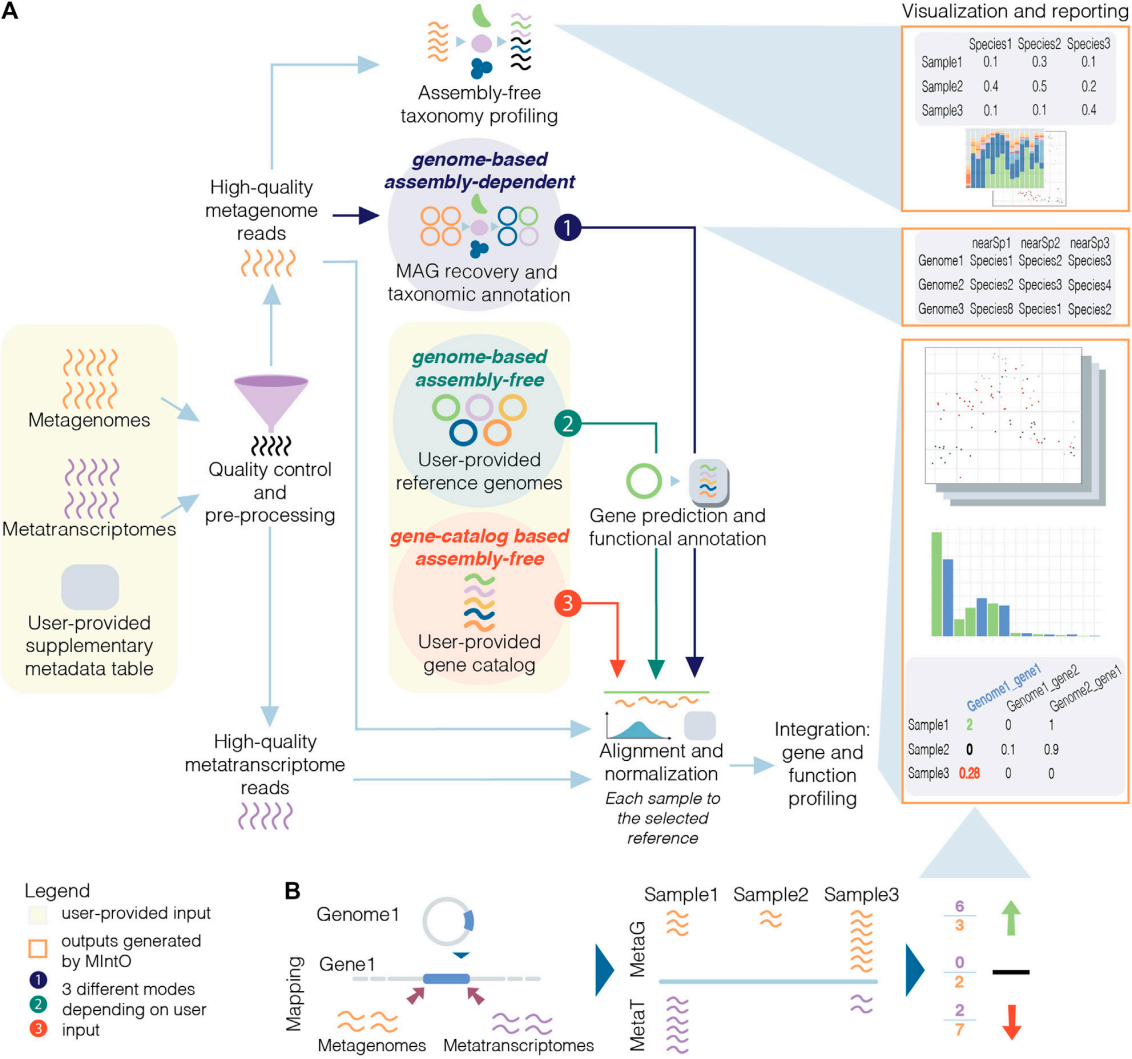
Schematic overview of metagenomic and metatranscriptomic integration to quantify gene expression levels. **(A)** Three modes are available based on the input data and the experiment design: the *genome-based assembly-dependent* mode (1, in dark purple) recovers MAGs from metagenomic samples, while the *genome-based assembly-free* (2, in dark green) and the *gene-catalog-based assembly-free* (3, in red) modes use publicly available genomes or a gene catalog, respectively, provided by the user. In the three modes, the pipeline workflow includes quality control and preprocessing; assembly-free taxonomy profiling of high-quality metagenomic reads (in orange) by identifying phylogenetic markers (coloured); alignment of the high-quality reads to the selected reference and normalization; integration: gene and functional profiling; and visualization and reporting. The gene prediction and functional annotation step is run using the recovered MAGs (mode 1) or publicly available genomes (mode 2). **(B)** The variation of gene expression depends on the abundance of transcripts from the organisms in the community and/or by changes in the abundance of these members and their related genes (community turnover).

Here, we introduce MIntO (Microbiome Integrated meta-Omics), a pipeline that includes state of the art tools to integrate microbiome metagenomic and metatranscriptomic data in a scalable way for read pre-processing, species composition profiling, MAG generation, gene and function expression profiling, as well as the visualization of the results and comparison of multiple samples. Optionally, MIntO can combine long-read sequences for more contiguous assemblies and short-read sequences for higher accuracy, which helps recover more accurate as well as complete MAGs (Bertrand et al., 2019; Overholt et al., 2020; Brown et al., 2021). Depending on the data availability and research question, the pipeline can be run in three modes: (A) *genome-based assembly-dependent*, (B) *genome-based assembly-free* and (C) *gene-catalog-based assembly-free* (Figure 1A).

MIntO enables the study of microbial ecology by linking functions to genomes and environmental context, helping to understand the dynamics of the molecular activities captured by the whole community-level changes in composition and gene expression (Figure 1B).

## Methods

MIntO v1.0.1 has been developed using R software (v4.0.3) (The R Project for Statistical Computing, 2021), Python 3 (Van Rossum and Drake, 2009) and Perl (Wall, Christiansen and Orwant, 2000) programming languages, and has been tested on a 64-bit Linux server with 2 × AMD EPYC 7742 64-Core Processors and 2 terabytes of memory.

### Conda Environment and Singularity Containers

MIntO has been designed to use publicly available software that are available as conda environments (Anaconda Inc, 2020) or singularity containers (Kurtzer, Sochat and Bauer, 2017) to minimize the installation of individual software packages by the user. All software dependencies are tied to specific versions in conda or singularity containers to ensure reproducibility and record-keeping of versions of the different libraries. It is encapsulated within a user-friendly framework using Snakemake (Mölder, 2021) to facilitate the scalability of the pipeline by optimizing the number of parallel processes from a single-core workstation to compute clusters. This pipeline enables consistency of the results and straightforward application by users with basic informatics skills to analyze complex omics data.

### Pipeline Inputs

MIntO requires a configuration file as an input indicating the metagenomic (metaG) and/or metatranscriptomic (metaT) sample names and the corresponding raw FASTQ files location together with the path of the pipeline dependencies, currently only FetchMGs (Kultima et al., 2012). MIntO generates the necessary directories and outputs the required files for further analysis, including the configuration files needed in each step of the pipeline, but they should be filled out by the user. Optionally, the required databases can be downloaded and installed by MIntO.

In addition, if MIntO is run under *genome-based assembly-free* mode, the user should provide input genomes as FASTA files, genome features as GFF files, and amino acid sequences of protein-coding genes as FASTA files, while in the case of *gene-catalog based assembly-free* mode the user should provide a multi FASTA file with the nucleotide sequences of the genes, such as the one published with the Integrated Gene Catalog (IGC) (Li et al., 2014) (Figure 1A, user-provided input).

### Pre-Processing of Metagenomic and Metatranscriptomic Short Reads

MIntO pre-processes metagenomic and metatranscriptomic short reads independently of each other. The pre-processing step can be subdivided into three different steps: quality and read length, host genome and ribosomal RNA (rRNA) filtering.

1. Quality and read length filtering.

We use Trimmomatic v0.39 (Bolger, Lohse and Usadel, 2014) to first remove sequencing adapters and low quality bases from raw reads and a second time to remove reads that are too short.

a. In the first step, the option *TRAILING:5 LEADING:5 SLIDINGWINDOW:4:20 ILLUMINACLIP:{adapters.fa}:2:30:10* is used if a sequence adapters file is provided by the user (*trimmomatic_adaptors = <PathTo>/adapters.fa*). Otherwise, a custom script retrieves the adapters by selecting the most abundant index in the first 10,000 headers of the raw FASTQ files (*trimmomatic_adaptors = False*). The user can decide to skip this step if adapter sequences have already been removed (*trimmomatic_adaptors = Skip*).
b. For the second filtering, the *MINLEN* parameter in Trimmomatic is used to remove reads that are too short. This cutoff is estimated as the maximum length above which a predefined percentage of the reads from the previous step are retained (default parameter is 95% of the reads, *perc_remaining_reads: 95*). If the estimated read length cutoff is below 50bp, trimmomatic will use 50bp as the minimum sequence length (Supplementary Figure S3).

2. Host genome filtering.

In the second step to remove putative host-derived sequences, the filtered read-pairs are aligned to a reference genome given by the user. The BWA aligner (Vasimuddin et al., 2019) version 2.2.1 is used to generate the index (*bwa-mem2 index*) and to map the read-pairs to the host genome (*bwa-mem2 mem -a*). Read-pairs aligned to this reference genome are identified by msamtools v1.1.0 (Arumugam, 2022) (*filter -S -l 30*) and excluded from the FASTQ files by mseqtools (https://github.com/arumugamlab/mseqtools) version 0.9.1, even if only one end is mapped (*subset --exclude --paired --list {listfile}*).

3. Ribosomal RNA filtering.

Prior to sequencing, it is recommended to deplete the rRNA in the metatranscriptomic samples. Nevertheless, it is common that metatranscriptomic sequence data still contains rRNA after such a depletion step. MIntO uses SortMeRNA v4.3.4 (Kopylova, Noé and Touzet, 2012) to map the metatranscriptomic reads to an rRNA sequence database consisting prokaryotic (16S and 23S) and eukaryotic (18S and 28S) rRNA sequences (*--paired_in --fastx --blast 1 --sam --other --ref*). Reads classified as rRNA by SortMeRNA are excluded from the FASTQ files using mseqtools (*subset --exclude --paired --list {listfile}*).

The remaining high-quality filtered (host-free for metagenomic and host- and rRNA-free for metatranscriptomic) reads are then passed to the sequence analysis and post processing steps.

### Assembly-Free Taxonomic Profiling From High-Quality Filtered Reads

High-quality filtered reads can be profiled by the default program, MetaPhlAn3 v3.0.13 (Beghini et al., 2021) (*--input_type fastq --bowtie2out -t rel_ab_w_read_stats*). Alternatively, users can choose to run mOTUs2 v2.1.1 (Milanese et al., 2019) in two different modes to generate a taxonomic profile as relative abundance (taxa_profile: *motus_rel*, *profile -u -q*) or as counts (taxa_profile: *motus_raw*, *profile -c -u -q*). If the latter one is chosen, MIntO estimates the relative abundance of the taxonomic profile. To explore the similarities and dissimilarities of the data, the relative abundance of the species composition is used to generate two visual outputs: 1) the 15 most abundant genera across the samples, and 2) a principal coordinate analysis (PCoA) using Bray-Curtis distance. These visualizations provide users with a general idea of the microbial composition in the different samples. For a more detailed downstream analysis, MIntO outputs the combined table of the taxonomy profiles of all samples in CSV format and as a phyloseq object (McMurdie and Holmes, 2013), the latter including the abundance of the species, taxonomic classification and metadata tables.

### Retrieving MAGs From Metagenomic High-Quality Host-Free Reads

MIntO’s approach to reconstruct MAGs from high-quality host-free reads exploits metagenomic assembly of single samples as well as co-assembly of pre-defined sample groups followed by binning preparation and contig binning.

1. Assembly:

a. Long-read assembly: If available, Nanopore reads are assembled individually using metaFlye assembler (Kolmogorov et al., 2020) v2.9 (*--nano-raw <FASTQ> --meta --min-overlap 3000 --iterations 3*)
b. Short-read assembly: MetaSPAdes assembler v3.15.3 (Nurk et al., 2017) is used to correct paired-end short reads from individual samples (*--only-error-correction,* the default *--phred-offset* is auto) followed by their single-assembly (*--meta --only-assembler*, the default kmer option is *k = 21,33,55,77,99,127*).
c. Hybrid assembly: Optionally, we can combine metagenomic Nanopore-based long reads and Illumina paired-end short reads to perform hybrid assembly by MetaSPAdes using the parameters as step (b) with an additional *--nanopore* option.
d. Co-assembly: MEGAHIT (Li et al., 2015) v1.2.9 is run with two different parameters (--*meta-sensitive* and *--meta-large*) per co-assembly, where by default all samples used in the single-assembly are assembled together. Users can also define their own subsets of samples that should be co-assembled in the configuration file.

2. Binning preparation:

Contigs longer than 2,500 bp from all the combinations of assemblies above are combined together in preparation for binning. Metagenomic reads from individual short-read metagenomes are first mapped to this set of contigs using BWA aligner (Vasimuddin et al., 2019) v2.2.1 (*bwa-mem2 mem -a*) in paired-end mode. Sequencing depth of the contigs in each sample is estimated by *jgi_summarize_bam_contig_depths* program included in MetaBAT2 (Kang et al., 2019).

3. Contig binning:

Contig binning is then performed by executing VAMB (Nissen et al., 2021), a binner using an unsupervised deep learning approach in the form of variational autoencoders that can be run with or without GPUs. GPU use is highly recommended if available in order to speed up the binning process, especially if working with a large number of samples. By default, MIntO runs VAMB four times, each time with a different set of parameters *-l 16 -n 256,256*; *-l 24 -n 384,384*; *-l 32 -n 512,512*; and -*l 40 -n 768,768*. However the user(s) can choose to perform just one run or a set of runs of their choice.

4. Non-redundant MAGs:

Bins generated by VAMB are split into MAGs derived from individual metagenomic samples. Only the MAGs that pass quality control using CheckM (Parks et al., 2015) (completeness > 90% and contamination < 5%) are kept. The MAGs are then subjected to cluster analysis performed with CoverM v0.6.0 (https://github.com/wwood/CoverM#usage, module cluster) in order to dereplicate them at 99% average nucleotide identity (ANI) (Jain et al., 2018). For each genome, a score is retrieved with the formula below.

assembly score=log10(longest contig length/#contigs)+log10(N50/L50) genome score=completeness−2∗contamination final score=0.1∗genome score+assembly score

Then for each cluster the genome with the highest score is chosen, generating a unique set of non-redundant MAGs which will be used in the next step.

### Taxonomic Assignment of MAGs

Once the unique set of MAGs is retrieved, taxonomy is assigned using the module *phylophlan_metagenomic* in PhyloPhlAn3 (Asnicar et al., 2020). MIntO uses SGB.Jul20 or SGB.Dec20 databases depending on user’s choice (*--database*) which will be automatically downloaded in the program folder if no other location is specified. Additionally, if the users have previously downloaded one of the PhyloPhlAn3 databases of their interest, they can use that by giving their path.

### Genome Annotation on the Retrieved MAGs

First, Prokka (Seemann, 2014) (version 1.14) (with options *--addgenes --centre X --compliant*) is used to identify and annotate the genes from the recovered MAGs, retrieving the corresponding nucleotide and amino acid sequences.

Next, predicted genes are annotated with several databases:

- eggNOG database (Huerta-Cepas et al., 2019) (COG ids) with eggNOG-mapper v2.1.6 (Huerta-Cepas et al., 2017; Cantalapiedra et al., 2021) (*--no_annot --no_file_comments --report_no_hits --override -m diamond* and *--annotate_hits_table -m no_search --no_file_comments --override*, emapperdb v5.0.2).
- KEGG functions (Kanehisa and Goto, 2000) (*-k -p prokaryote.hal --create-alignment -f mapper*, Kofam_scan (Aramaki et al., 2020) version 1.3.0 and ko_list from November 2021).
- Carbohydrate-active enzyme database [CAZyme, (Huang et al., 2018; Zhang et al., 2018)] with dbCAN annotation tool v2.0.11 (Zhang et al., 2018) (*run_dbcan.py protein*).
- Pfam database (Mistry et al., 2021) with eggNOG-mapper (Huerta-Cepas et al., 2017; Cantalapiedra et al., 2021).

These databases are installed locally by the user. The pipeline integrates the different gene annotations: Gene ID, eggNOG, KEGG_ko, KEGG_Pathway, KEGG_Module, dbCAN.mod, dbCAN.enzclass and Pfam.

### Functional Profiling

The high-quality filtered (host-free for metagenomic and host- and rRNA-free for metatranscriptomic) reads are used to generate the functional profiles following four steps: metagenomic and metatranscriptomic read alignments, mappability ratio, read count normalization, and gene and function expression computation.

#### Metagenomic and Metatranscriptomic Reads Alignment

To estimate gene and transcript abundances, the high-quality filtered reads can be aligned to 1) genomes such as the recovered MAGs or publicly available genomes (*genome-based*) or 2) a gene catalog (*gene-based*), depending on the mode that the pipeline is run.

1. *Genome-based* alignment: The retrieved MAGs or the reference genomes are concatenated and indexed using the BWA aligner (Vasimuddin et al., 2019) v2.2.1 (*bwa-mem2 index*). Mapping reads to the reference (*bwa-mem2 mem -a*) is followed by highest-scoring alignment(s) filtering for each read with msamtools v1.1.0 (Arumugam, 2022) (*filter -S -b -l 50 -p 95 -z 80 --besthit*)*.* The filtered BAM files are indexed by samtools v1.14 (Danecek et al., 2021) (*sort --output-fmt = BAM*; *index*) and the GFF file with the genome features is used to quantify the raw number of aligned reads to each gene by bedtools *multicov* v2.29.2 (Quinlan and Hall, 2010).
2. *Gene-based* alignment: As an alternative, the gene catalog given by the user is indexed using *bwa-mem2 index* [BWA aligner v2.2.1 (Vasimuddin et al., 2019)]. The aligned reads (*bwa-mem2 mem -a*) are filtered for highest-scoring alignment(s) per read with msamtools v1.1.0 (Arumugam, 2022) (*filter -S -b -l 50 -p 95 -z 80 --besthit*)*.*

Optionally, the user can filter the aligned reads by establishing the minimum number of mapped reads to a gene, using the *MIN_mapped_reads* parameter. While the default value for this parameter is 0, for metagenomes with sequencing depth higher than 10 million paired-end reads, we recommend setting this threshold at 10 mapped reads to a gene (MIN_mapped_reads: 10), which is what we used for IBDMDB dataset.

#### Mappability Ratio

In addition, to estimate how representative the gene or genome databases are of the metagenomic and metatranscriptomic samples, the filtered BAM files are used to calculate the mappability ratio by msamtools v1.1.0 (Arumugam, 2022) (*profile --total {total_reads} --multi prop --unit all --nolen*). Here, we used the IGC (Li et al., 2014) and recovered MAGs as references.

#### Read Count Normalization

Normalization of read counts makes possible the comparison within or between different samples. Based on the users’ selection, TPM (Transcripts Per Kilobase Million) or MGs (Marker Genes) normalized gene and transcript abundance profiles are generated from the metagenomic and metatranscriptomic read alignments, respectively.

1. *TPM normalization.* Sequencing depth and gene length are used to obtain the relative abundance of genes or transcripts (Wagner, Kin and Lynch, 2012). The TPM value of the gene *i*, TPM(*i*), is calculated by employing the equation:

$$TPM\left(i\right)=\;\frac{reads\;mapped\;to\;gene/gene\;length}{\;\;sum\left(reads\;mapped\;to\;gene/gene\;length\right)\;}\;\times \;10^{6}\;=\;\frac{n_{i}/l_{i}}{{\text{ Σ}}_{j}n_{j}/l_{j\;}}\times \;10^{6}\;$$

 where *n*_*i*_ is the number of reads mapped to the gene *i*, *l*_*i*_ is the length of that gene and *j* iterates over all genes identified in the sample.

2. *MGs normalization.* In a similar approach to Salazar et al. study, but more customized to MAG-based analysis, the gene or transcript abundances of a MAG are divided by the median abundance of 10 universal single-copy phylogenetic MGs from the corresponding MAG (Salazar et al., 2019). These MGs are identified in each MAG by FetchMGs v1.2 (available at http://motu-tool.org/fetchMG.html) as OGs: COG0012, COG0016, COG0018, COG0172, COG0215, COG0495, COG0525, COG0533, COG0541, and COG0552. In addition, these MGs are constitutively expressed housekeeping genes across many different conditions (Sunagawa et al., 2013; Milanese et al., 2019; Salazar et al., 2019). Thus, the MGs-normalized metagenomic and metatranscriptomic profiles can be interpreted as the gene and transcript abundances in a MAG relative to housekeeping MGs abundance and transcript, respectively. The MGs value of the gene i, MGs(i), is calculated by employing the equation:

$$MG\left(i\right)=\;\frac{\;\;reads\;mapped\;to\;gene/gene\;length\;\;}{\;\;median\;10\;MGs\;from\;a\;genome\;}\;\times \;10^{6}\;=\;\frac{n_{i}/l_{i}}{\;\;M\left(MGs\right)\;}\times \;10^{6}$$

where *n*_*i*_ is the number of reads mapped to the gene *i* in the gene’s MAG, *l*_*i*_ is the length of that gene and *M*(*MGs*) is the median abundance of the 10 MGs from the gene’s genome.

When the reads are mapped to a gene database, msamtools v1.1.0 (Arumugam, 2022) is used to normalize the number of aligned reads per gene to TPM (*profile --total {total_reads} --multi prop --unit tpm*). However, if the reads are mapped to a set of MAGs or publicly available genome(s), the user can choose to obtain TPM or MGs normalized abundances.

#### Computing Gene and Function Expression Profiles

The levels of gene expression are computed by the integration of gene and transcript abundance profiles, which is, the relative amount of RNA molecules per DNA copy of that gene (TPM normalization):

gene expression=transcript abundance/gene copy number

Or gene expression in that MAG relative to housekeeping MGs expression (MGs normalization):

MGs‐normalized gene expression=gene expression /median MGs gene expression

Finally, functional profiles are obtained by grouping the genes into functions.

### Visualization

All the visualization outputs are generated in R software (v4.0.3) (The R Project for Statistical Computing, 2021), using the following packages: BiocManager (v1.30.16) (Morgan, 2021), data.table (v1.14.2) (Dowle and Srinivasan, 2021), reshape2 (v1.4.4) (Wickham, 2007), phyloseq (v1.34.0) (McMurdie and Holmes, 2013), tidyverse (v1.3.1) (Wickham et al., 2019), ggplot2 (v3.3.5) (Wickham, 2016), ggrepel (v0.9.1) (Wickham, 2007; Slowikowski, 2021), dplyr (v1.0.7) (Wickham et al., 2021), tidyr (v1.1.4) (Wickham and Girlich, 2021), stringr (v1.4.0), rlang (v0.4.11) (Henry and Wickham, 2021), haven (v2.4.3) (Wickham and Miller, 2021), vegan (v2.5-7) (Oksanen et al., 2020), keggrest (v1.30.1) (Tenenbaum, 2017), and pfam.db (v3.12.0). To have a better representation of the result, it is recommended to provide a metadata table by including the file path in the config file (*METADATA*) with sample ID, conditions and sample alias columns. If no metadata are provided, the sample IDs are used to generate the plots. However, the user can always use MIntO outputs for further downstream analysis.

### Data

#### Inflammatory Bowel Disease Multi’Omics Database Samples

We used 91 human fecal metagenomes from the Inflammatory Bowel Disease Multi’omics Database [IBDMDB, (Lloyd-Price et al., 2019)]. The IBDMDB study provides matching Illumina metagenomic and metatranscriptomic data. We selected six participants diagnosed as non-IBD [P6018 (nIBD1), M2072 (nIBD2)]; Crohn’s disease [H4006 (CD1) and H4020 (CD2)]; and ulcerative colitis [H4019 (UC1) and H4035 (UC2)] that were followed for 1 year each (Supplementary Table S1). Sample H4019_20 was not included due to a parsing error. Sequence data were retrieved from NCBI Short Read Archive under BioProject identifier PRJNA398089.

#### Paired-End Illumina and Nanopore-Based Metagenomic Data From Head and Neck Cancer Patients

We used human fecal metagenomes from head and neck cancer (HNC) patients (Wongsurawat et al., 2019), where samples were sequenced using Illumina and Nanopore technologies. We selected a subset of five patients: PatientHNC_03, PatientHNC_05, PatientHNC_06, PatientHNC_08 and PatientHNC_10. These were obtained from NCBI Short Read Archive under the accession numbers SRR7947170, SRR7947175, SRR7947177, SRR7947178, SRR7947179, SRR7947181, SRR7947184, SRR7947185, SRR7947186 and SRR7947187.

#### Human Genome

During MIntO pre-processing, the human genome (build hg38) was used to remove putative host-derived sequences (host genome filtering step).

### Implementation of the Pipeline

MIntO implementation and automation are achieved by Snakemake (Mölder, 2021), a user-friendly framework that facilitates the scalability of the pipeline by optimizing the number of parallel processes from a single-core workstation to compute clusters. MIntO leverages singularity containers (Kurtzer, Sochat and Bauer, 2017) and Conda environments (Anaconda Inc, 2020) to ensure version control of the different libraries and implements a pipeline connecting several state of the art bioinformatic tools. In this way, MIntO enables consistency of the results and straightforward application by users with basic informatics skills to analyze complex omics data. The only dependencies are FetchMGs and Conda.

## Results

MIntO can be run in three different modes, thanks to its modular design, depending on the user’s preference and available data: *genome-based assembly-free*, *gene-catalog-based assembly-free* and *genome-based assembly-dependent*. For all the three modes, users have to input FASTQ files from metagenomic and/or metatranscriptomic paired-end raw short reads and optionally, nanopore-based long reads, as well as a configuration file indicating the metagenomic and/or metatranscriptomic sample names and the corresponding location of raw FASTQ files. In the *genome-based assembly-dependent* mode, the given metagenomes are used to retrieve MAGs, while in the two *assembly-free* modes, *genome-based* or *gene-catalog-based*, the user also has to provide a set of reference genomes or a gene-catalog database, respectively, to generate the gene and functional profiles. These two options could be used when the user is working with a defined community or when there are not enough metagenomic samples to generate representative MAGs. These three modalities are illustrated in Figure 1A.

MIntO can be divided into seven major steps, which will be discussed in the next paragraphs using our analysis of example data (Figure 1A):

1. Quality control and pre-processing
2. Assembly-free taxonomy profiling
3. Recovery of MAGs and taxonomic annotation (only run in *genome-based assembly-dependent* mode)
4. Gene prediction and functional annotation (only run in *genome-based* modes)
5. Alignment and normalization
a. g*enome-based* mode: recovered MAGs or publicly available genomes
b. *gene-based* mode: gene catalog
6. Integration: Gene and functional profiling
7. Visualization and reporting

The third step is skipped if an assembly-free mode is selected, and the fourth step is skipped when *gene catalog-based assembly-free* mode is chosen (Figure 1A). An overview of the directories generated can be seen in Supplementary Figure S1 *.*

To illustrate the use of MIntO, a set of 91 human fecal metagenomes from the Inflammatory Bowel Disease Multi’omics Database (IBDMDB) was selected (Lloyd-Price et al., 2019). These samples correspond to six participants diagnosed as non-IBD (nIBD1 and nIBD2), Crohn’s disease, (CD1 and CD2) and ulcerative colitis, (UC1 and UC2), which were followed for 1 year each (Supplementary Figure S2, Supplementary Table S1). The IBDMDB study provides matching Illumina metagenomic and metatranscriptomic data. The subset of samples used here correspond to 933.4 and 612 million read-pairs (2 × 101 bp) from metagenomic and metatranscriptomic sequencing, respectively (mean 10.85 million read-pairs, ranging from 0.26 to 21.04 million for metagenomic; mean 6.18 million read-pairs, ranging from 0.01 to 15.72 million for metatranscriptomic).

Here, we present the results from the *genome-based assembly-dependent* and *gene catalog-based assembly-free* modes, where we used recovered MAGs and the Integrated Gene Catalog (IGC) (Li et al., 2014), respectively, as reference to profile genes and functions.

### Quality Control and Pre-Processing

The IBDMDB dataset was already filtered by quality and sequence adapters, therefore the first step in the pre-processing of the 91 samples was skipped (*trimmomatic_adaptors = Skip*, see Methods). We then used a minimum read length cutoff of 53 bp for metagenomic and 54 bp for metatranscriptomic to keep 95% of the longest sequences using Trimmomatic (Bolger, Lohse and Usadel, 2014) (Supplementary Figure S3).

Subsequently, putative host-derived sequences were removed using the human genome (build hg38). In silico rRNA sequences screening was exclusively applied to metatranscriptomic reads using SortMeRNA (Kopylova, Noé and Touzet, 2012). This resulted in a total number of 599.4 million high-quality read-pairs for metagenomic and 910.9 million high-quality read-pairs for metatranscriptomic data (Table 2, Supplementary Figure S4).

**TABLE 2:** Median (minimum and maximum) of raw and high-quality million read-pairs in the 91 human fecal microbiome samples from the IBDMDB.

	metagenomic	metatranscriptomic
Raw read-pairs (millions)	10.85 (10.15–21.04)	6.18 (6.65–15.72)
High quality read-pairs (millions)	10.56 (9.9–20.58)	6.04 (6.52–15.45)

### Assembly-Free Taxonomy Profiling

**FIGURE 2:**
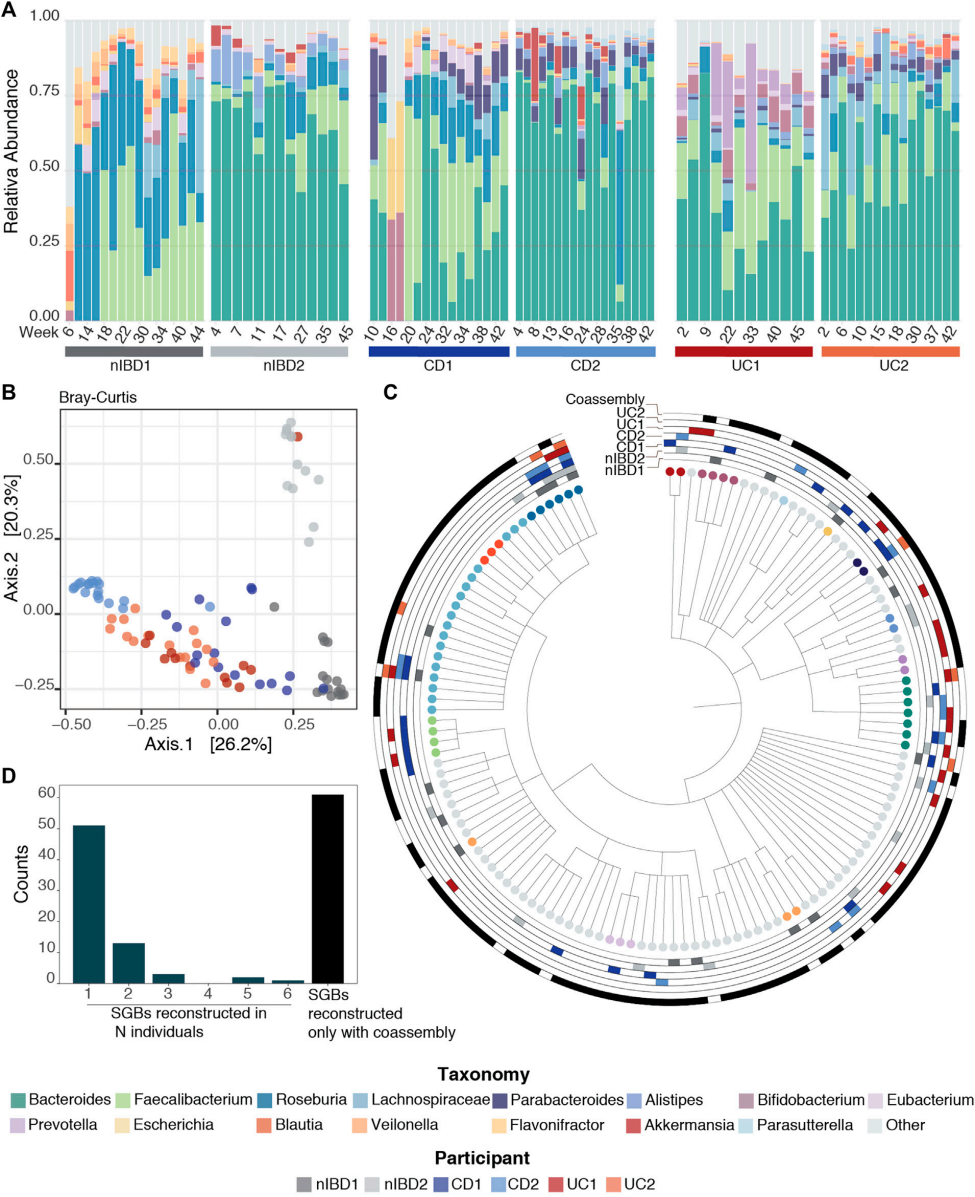
Taxonomic profiles. **(A)** Relative abundance for the 91 samples for the 15 most abundant genera across the samples using MetaPhlAn3 (Beghini et al., 2021). **(B)** Projection of the first two principal coordinates based on Bray–Curtis dissimilarity from the microbiome composition using MetaPhlAn3 (Beghini et al., 2021). **(C)** Taxonomy tree representing the 131 SGBs taxonomies after running PhyloPhlAn3 (Asnicar et al., 2020) on the retrieved MAGs. The first six rings mark MAGs that were retrieved in the 6 patients with the different conditions used in this work (nIBD, CD and UC), while the last ring marks the MAGs obtained from co-assembly. **(D)** Distribution of the SGBs in the 6 patients: 51 SGBs taxonomies were retrieved from just one sample, 13 from two samples, 3 from three samples, 2 from five samples and 1 in all the samples. The last bar represents the 61 taxonomies that were found only by having performed co-assembly.

Once the reads were pre-processed, high-quality reads were profiled at species level using MetaPhlAn3 (Beghini et al., 2021) (Figure 1A, assembly-free taxonomy profiling step). In Figure 2A, we can see the temporal shifts and dynamics exhibited by microbes over the course of 1 year and the difference of microbial composition between the six participants focusing on the 15 most abundant genera across the samples. In general, the most predominant genera are *Bacteroides*, *Faecalibacterium* and *Roseburia*. The constitution of a separate cluster by samples from participant nIBD2 in Figure 2B cannot be explained by the 15 most abundant genera across the samples (Figure 2A), but it could be due to the difference in composition of lower-abundance bacteria.

### Recovery of MAGs and Taxonomic Annotation

In parallel, the pre-processed reads underwent the assembly step in the *genome-based assembly-dependent* mode (Figure 1A, recovery of MAGs and taxonomic annotation step). As this dataset consists of short-read metagenomes only, we used two assembly approaches to recover high-quality scaffolds: 1) assembly of each metagenome individually (single-assembly) using MetaSPAdes assembler (Nurk et al., 2017) and 2) assembly of all metagenomes together (co-assembly) using MEGAHIT (Li et al., 2015) assembler. Genome bins were generated from assembled scaffolds that were at least 2,500 bp long by mapping the 91 samples individually to the scaffolds, calculating the sequence depth of each scaffold in the 91 samples, and finally running VAMB (Nissen et al., 2021) four times with different parameters and GPU mode (see Methods).

After binning, 5,048 MAGs were retrieved from the 91 metagenomic samples. Using CheckM (Parks et al., 2015), we identified high-quality (HQ) MAGs (completeness > 95% and contamination < 5%) and kept 957 MAGs. We then obtained unique high-quality MAGs when clustering the HQ MAGs at 99% ANI distance (Jain et al., 2018) with CoverM (https://github.com/wwood/CoverM#usage) and choosing the best genome in a given cluster using a genome quality score (see Methods). This de-replication process resulted in 163 MAGs which constituted a set of non-redundant genomes (available at 10.5281/zenodo.6360083). These MAGs are useful to collectively explain the ecological description and biodiversity in the samples, and to capture sample-specific variation at functional and abundance level without relying on publicly available reference genomes. Additionally, working with a restricted number of genomes is helpful to speed up the next steps of the pipeline.

The taxonomic annotation of the 163 MAGs was performed by *phylophlan_metagenomic* module in PhyloPhlAn3 (Asnicar et al., 2020), which also provides taxonomic lineage information about the 10 nearest genomes in the PhyloPhlAn3 genome database. Each MAG was assigned to a species-level genome bin (SGB) if its closest genome in the database was within 5% average nucleotide identity. This resulted in the 163 MAGs falling into 131 SGBs (Figure 2C). In general, MAGs with a distance higher than 5% to the closest genome in the database can be considered as putative novel species (Manara et al., 2019; Pasolli et al., 2019). However, we did not recover any MAGs from putative novel species in this dataset.

By default, MIntO performs co-assembly, which although time consuming, is an extremely important step. In fact, we obtained the highest number of unique taxa from the co-assembled samples compared to any single-sample assembly (Table 3). Remarkably, 61 of the 131 taxonomies (∼46%) could be retrieved only by performing co-assembly (Figure 2D). With single-sample assembly we still retrieved 31 (∼23%) unique taxonomies not covered by the co-assembled samples, of which 13 (∼10% of the total) are only found in one sample (Figure 2C). This is helpful to better distinguish sample-specific composition, as for example *Akkermansia muciniphila* SGB9228, which is the second *Akkermansiacae* species by presence in the human population (Karcher et al., 2021) can only be found in patient CD1. These results are achievable only by performing both single and co-assembly.

**TABLE 3:** Number of SGB taxonomies retrieved per sample.

Sample/Method	Number of Taxa
nIBD1	18
nIBD2	21
CD1	24
CD2	15
UC1	21
UC2	24
Co-assembly	100

In addition, we performed our own benchmark to show that combining long and short reads improves the assembly contiguity. MIntO assembled paired-end metagenomes from the gut microbiota of five patients with head and neck cancer (Wongsurawat et al., 2019), which were generated by 1) Illumina-only, or 2) Illumina and Nanopore sequencing platforms. The number of generated scaffolds (127,315 and 172,888 for Illumina and Illumina + Nanopore, respectively), and their mean length (9.44 kb and 9.72 kb for Illumina and Illumina + Nanopore, respectively), were greater when long-reads were included in the assembly. Furthermore, Illumina + Nanopore assembly generated 13 scaffolds longer than 600 kb with a maximum of 1,119 kb, whereas the assembly of Illumina-only data generated 2 scaffolds longer than 600 kb with a maximum of 736 kb. Finally, the scaffold length distribution shows that scaffolds from Illumina + Nanopore assemblies are more contiguous than Illumina-only assemblies (Supplementary Figure S6).

### Gene Prediction and Functional Annotation

**FIGURE 3:**
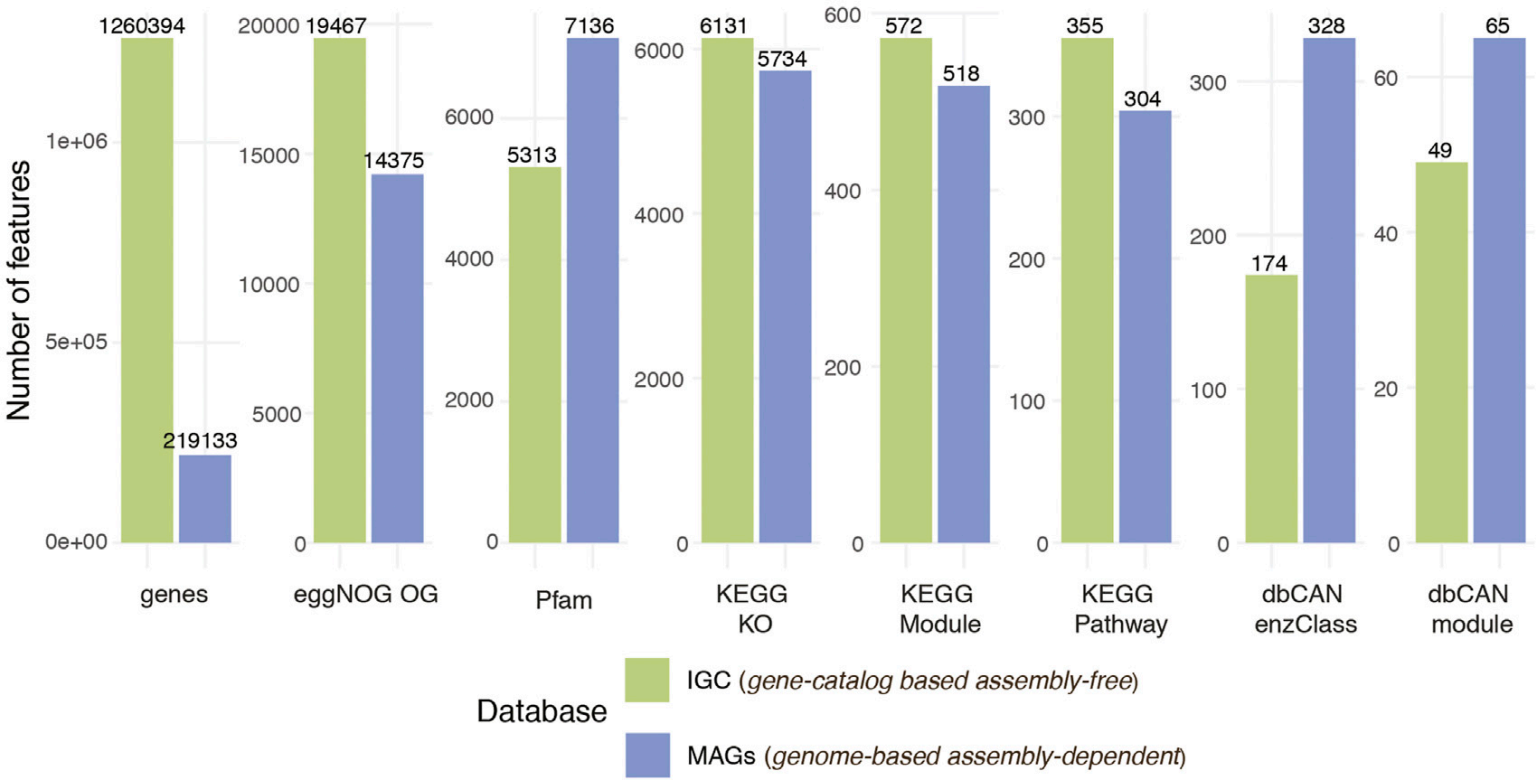
Comparison of number of genes and features per function database between non-redundant high quality 163 MAGs and IGC.

The unique set of MAGs recovered in the previous step underwent gene prediction and functional annotation (Figure 1A, gene prediction and functional annotation). Prokka (Seemann, 2014) was used to identify and annotate the genes, retrieving the corresponding nucleotide and amino acid sequences. A total of 412,394 genes were predicted in the 163 recovered MAGs. These were annotated with seven different functional databases: eggNOG (Yin et al., 2012; Huerta-Cepas et al., 2019), KEGG Pathways, Modules and KOs (Kanehisa and Goto, 2000), dbCAN modules and enzyme classes (Yin et al., 2012), and Pfam (Mistry et al., 2021) (Figure 3). The same process could also be applied to user-provided genome sequences under *genome-based assembly-free* mode.

The gene and function annotation step was skipped in the *gene catalog-based assembly-free* mode as we used existing eggNOG, KEGG Pathways, KEGG Modules, KEGG KO, dbCAN modules, dbCAN enzymes class, Pfam function annotation for IGC (available at https://db.cngb.org/microbiome/genecatalog/genecatalog_human/). The number of expressed genes and functions for both modes are summarized in Figure 3. Even though we detected > 5 × genes by mapping the metagenomes to IGC compared to genes encoded in the 163 MAGs, genes from the MAGs covered the vast majority of the functions detected via IGC. In some cases such as Pfam and CAZy databases, MAGs recovered more functions suggesting that contiguous assemblies and more complete genes could improve the quality of functional annotations.

### Alignment and Normalization

The metagenomic and metatranscriptomic high-quality reads were mapped to a reference database followed by TPM normalization to obtain the relative abundance of genes from metagenomic read alignments (i.e., gene abundance profile) and transcripts from metatranscriptomic read alignments (i.e., gene transcript profile) (Figure 1A, alignment, normalization and integration). We used as a reference database the 163 recovered MAGs for the *genome-based* mapping and the IGC (Li et al., 2014) for the *gene-based* alignment. Overall, the mappability rate at 95% of sequence identity for MAGs (median 72.26%) was lower than for IGC (median 92.47%) with the highest difference for participant CD2 (Supplementary Figure S5), which could be due also to the lower number of taxonomies retrieved for the samples (Table 3). However, this difference was not as remarkable when using metatranscriptomic reads (77.61 and 73.9% median, respectively).

### Integration: Gene and Function Expression Profiling

The variation of microbial community transcript levels may be affected by the changes in gene expression and/or by the community turnover. To disentangle the individual contributions of these mechanisms across the different samples, we integrated gene abundance and transcript abundance profiles (Salazar et al., 2019) (see Methods). The obtained levels of gene expression represent the relative amount of expressed transcripts per gene (Figure 1A, integration: gene and functional profiling). From the 412,394 predicted genes in the 163 recovered MAGs, 219,133 genes were expressed in at least one sample, while we detected the expression of 1,260,394 genes from the 9.9 million genes in IGC.

Furthermore, the corresponding gene profiles were used to generate the function abundance, transcript and expression profiles by grouping the annotated genes into functions. The highest number of features detected in the samples corresponded to the eggNOG database on both modes, followed by Pfam or KEGG KO (Figure 3). We identified 5,734 and 6,131 KEGG KO expressed features when we used the recovered MAGs and IGC as a reference, respectively. Among the 7,217 KEGG KO functions identified between the two profiles, 64.4% (4,651 features) were found in both. The 15% of features (1,086) uniquely identified in the MAGs could correspond to genomes not included in the database and the 20.5% of the functions (1,481) detected in IGC could belong to low abundant bacteria whose genomes could not be retrieved or were missed due to MAGs filtered out based on our quality criteria.

**FIGURE 4:**
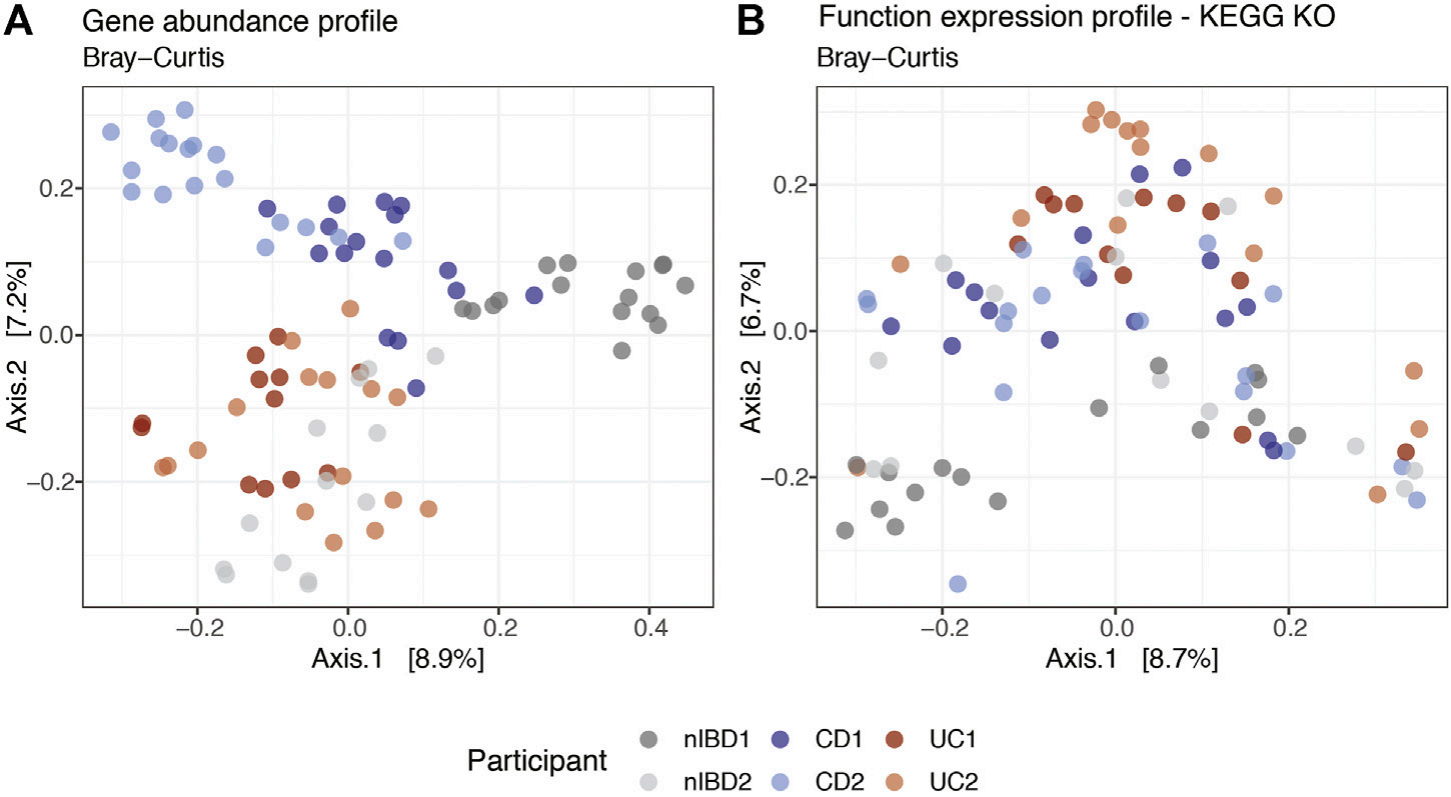
Projection of the first two principal coordinates based on expression profiles Bray–Curtis dissimilarity at **(A)** gene and **(B)** function KEGG KO levels using a subset of 91 samples from IBDMDB. Labels correspond to the *sample alias* and are colored by *condition* (patients diagnosis).

We used MIntO’s visualization features to perform principal coordinate analysis (PCoA) on the different gene and functional profiles to observe the longitudinal compositional changes and to compare the dissimilarities between participants. In Figure 4A we show the gene expression PCoA plot for the *assembly-free gene catalog* mode using IGC (Li et al., 2014). In general, the samples were clustered by Crohn’s disease and Ulcerative colitis diagnosis suggesting a similar bacterial abundance and expressed genes due to the presence of the disease (Kostic, Xavier and Gevers, 2014; Lloyd-Price et al., 2019). Samples from participants used as control (nIBD1 and nIBD2) were clustered separately, probably due to the inter-individual variations in the microbiome composition. In fact, the most abundant genus in all participants was *Bacteroides*, with the exception of nIBD1 where *Roseburia* and *Faecalibacterium* were predominant. At transcript level (Supplementary Figure S7), the dissimilarity between the samples explained by the first two principal coordinates (18.7% and 12.2%) was higher than at gene expression level (8.9% and 7.2%). The transcript abundance changes might be mainly attributed either to differences in the expression of genes encoded by the microbes in the community or changes in the abundance of these members and their related genes or a combination of these mechanisms. Hence, the computation of gene expression profiles by the integration of abundances of genes and the respective transcripts is of crucial importance to obtain a more accurate representation of ecologically relevant processes that are occurring.

Overall, the dissimilarities between the samples were visible at the gene expression, gene abundance and transcript abundance profiles (Figure 4A and Supplementary Figure S7). However, at function expression level (Figure 4B) the clusters were not as well defined, suggesting that genes from different species could harbor the same functions in different microbial communities. Although the taxonomic composition differed between the six participants and consequently the gene composition and expression, the functional profiles across individuals and time were more conserved (functional redundancy) (Tian et al., 2020). Differences in functional profiles between nonIBD and IBD diagnosed participants could provide insights into the functions involved in microbiome–host interactions at states of health or disease (Heintz-Buschart and Wilmes, 2018).

### Visualization and Reporting

Further analyses can be done using the output files (Figure 1A, visualization and reporting; Supplementary Figure S1). MIntO generates three different types of table: 1) assembly-free and assembly-based taxonomic profiles; 2) gene profiles, including the gene IDs [generated by Prokka (Seemann, 2014; Beghini et al., 2021) when selecting *assembly-dependent* mode or sequence IDs when choosing *assembly-free* mode] and normalized gene abundance, transcript or expression; and 3) functional profiles per database, including the function IDs, function description and function abundance, transcript or expression normalized counts. For an easier downstream analysis of these data, phyloseq objects are generated for the taxonomic, gene and functional profiles.

MIntO also outputs the shown plots as preliminary results to help the user in the downstream analysis (Figures 2A,B, Figures 3, 4, Supplementary Figures S3, S7).

The metadata provided in IBDMDB (Supplementary Table S1) was given as an input to the pipeline, which colored the samples by *sample_alias* (participant’s ID) in the output plots.

## Discussion

MIntO is a versatile pipeline that integrates metagenomic and metatranscriptomic data, beyond a comparison of the gene and transcript abundances, in order to quantify gene and function expression in a very straightforward way. The modular design of MIntO enables the user to run the pipeline using three available modes based on the input data and the experimental design.

In order to illustrate the pipeline, a subset of 91 human fecal microbiome samples from the IBDMDB (Illumina metagenomic and metatranscriptomic paired-reads) was used to run the full version of the pipeline with default parameters. Here, we show the complementary results from two of the three available modes, *genome-based assembly-dependent* and *gene catalog-based assembly-free*. In the former, MIntO retrieved 163 high-quality non-redundant MAGs that encoded 412,394 genes, among which 219,133 genes were expressed in at least one sample, while 1,260,394 genes from IGC were expressed in the *gene catalog-based assembly-free* mode. Overall, the dissimilarities between the samples were visible at the taxonomic and gene levels, while the functional profiles across individuals and time were more conserved (functional redundancy), indicating that strain-specific genes from different microbiomes represented similar functions. Interestingly, among the 7,217 KEGG KO functions identified between the two profiles, 15% of the features were uniquely identified in the MAGs and 20.5% of the functions were detected in IGC.

The distinctive feature of this pipeline is the integration of the metagenomic and metatranscriptomic data, to obtain the expression profiles and furthermore the functional profiles by annotating the sequences with several databases. This enables us to study in detail the variation in expression of the genes and functions in the different samples across time and experiment conditions, thus the community behavior. Overall, the IBDMDB-samples clustered by the participant ID using the genes and transcript abundances and gene expression. However, using the KEGG KO annotations at function expression level, the clusters are not as well defined, due to the functional redundancy (Tian et al., 2020).

Another important feature of MIntO is performing *de novo* assembly and contig binning to recover high-quality MAGs from metagenomic reads, which compared to other methods utilizes an accurate unsupervised deep learning approach in the form of variational autoencoders (Nissen et al., 2021). The *assembly-dependent* mode could be helpful to retrieve novel genomes that are missed by reference-dependent profiling methods (Pasolli et al., 2019). The recovery of MAGs is indispensable to uncover the diversity of bacteria in an environment and it is crucial for an optimal calculation of the variation of gene expression, including unknown or functional genes from biosynthetic gene clusters (Youngblut et al., 2020). Additionally, new putative genomes can increase the number of known species in the available databases, especially when the analyses are performed on metagenomes coming from new environmental sources.

In conclusion, in this paper we show how MIntO can be a useful tool to analyze metagenomic and metatranscriptomic data in a standardized way, enabling the study of microbial ecology by linking functions to genomes and environmental context. We foresee that this pipeline will contribute to the understanding of the dynamics of the molecular activities captured by the community turnover and gene expression alterations as the cause that shapes community transcript levels. Elucidating the functions and characterizing the specific strains of a community will be crucial to increase our knowledge of the microbiome’s contribution to human health and environment.

## Data Availability

Publicly available datasets were analyzed in this study. Matching Illumina metagenomic and metatranscriptomic data from IBDMDB can be found here: https://ibdmdb.org/tunnel/public/summary.html, IBDMDB, BioProject identifier PRJNA398089. Matching shotgun metagenomic data generated from both Illumina and Nanopore technologies can be found in NCBI Short Read Archive under the accession numbers SRR7947170, SRR7947175, SRR7947177, SRR7947178, SRR7947179, SRR7947181, SRR7947184, SRR7947185, SRR7947186 and SRR7947187. Non-redundant MAGs constructed by MIntO from 91 metagenomes from IBDMDB are available at https://doi.org/10.5281/zenodo.6360083.
