# Supplementary figures and images for: MIntO: A Modular and Scalable Pipeline For Microbiome Metagenomic and Metatranscriptomic Data Integration
Source: Front Bioinform. 2022 May 10;2:846922. doi: 10.3389/fbinf.2022.846922 (PMC9580859; doi:10.3389/fbinf.2022.846922)

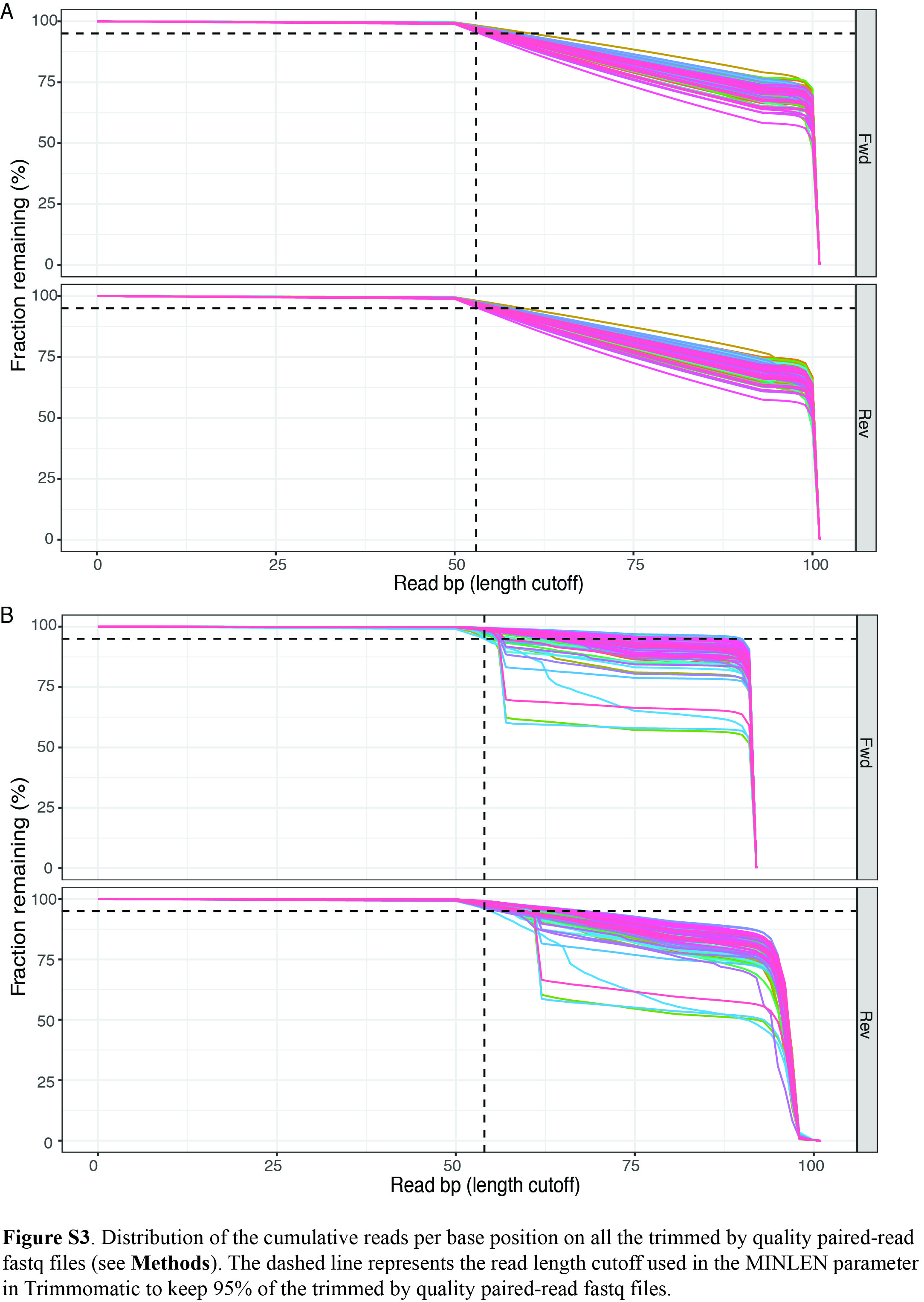

Supplement: Supplementary file 2 [file Image3.JPEG]

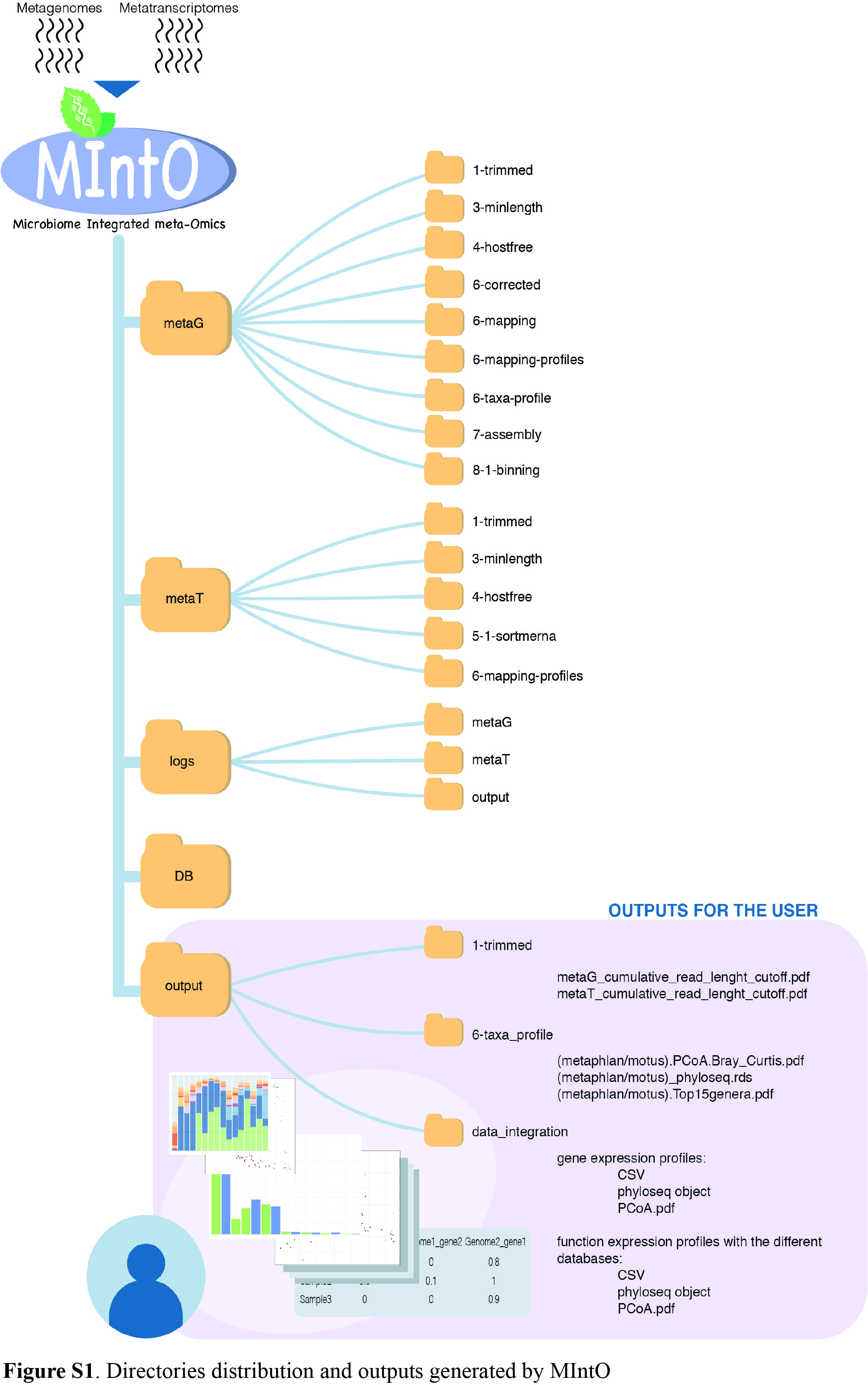

Supplement: Supplementary file 4 [file Image1.JPEG]

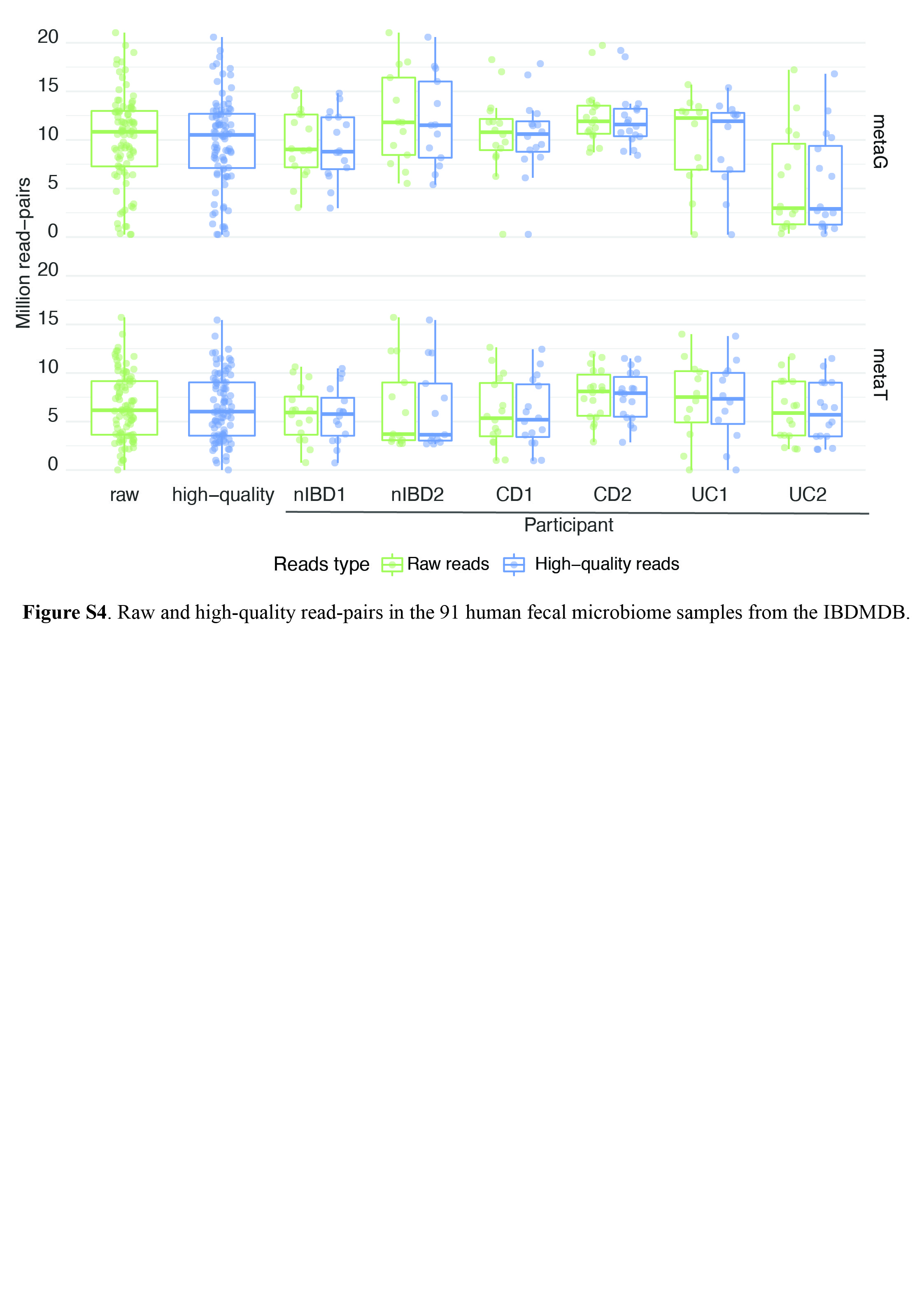

Supplement: Supplementary file 5 [file Image4.JPEG]

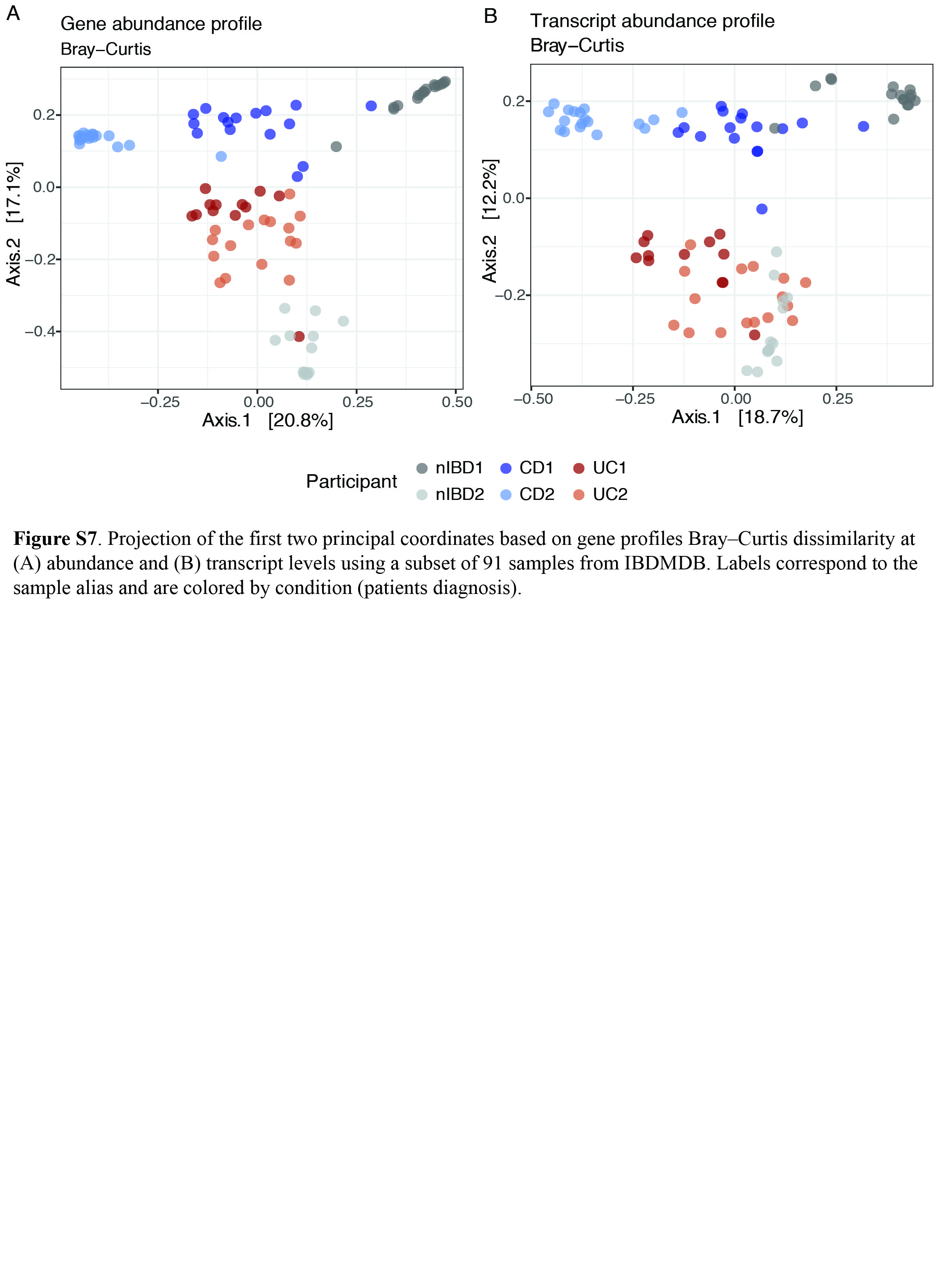

Supplement: Supplementary file 6 [file Image7.JPEG]

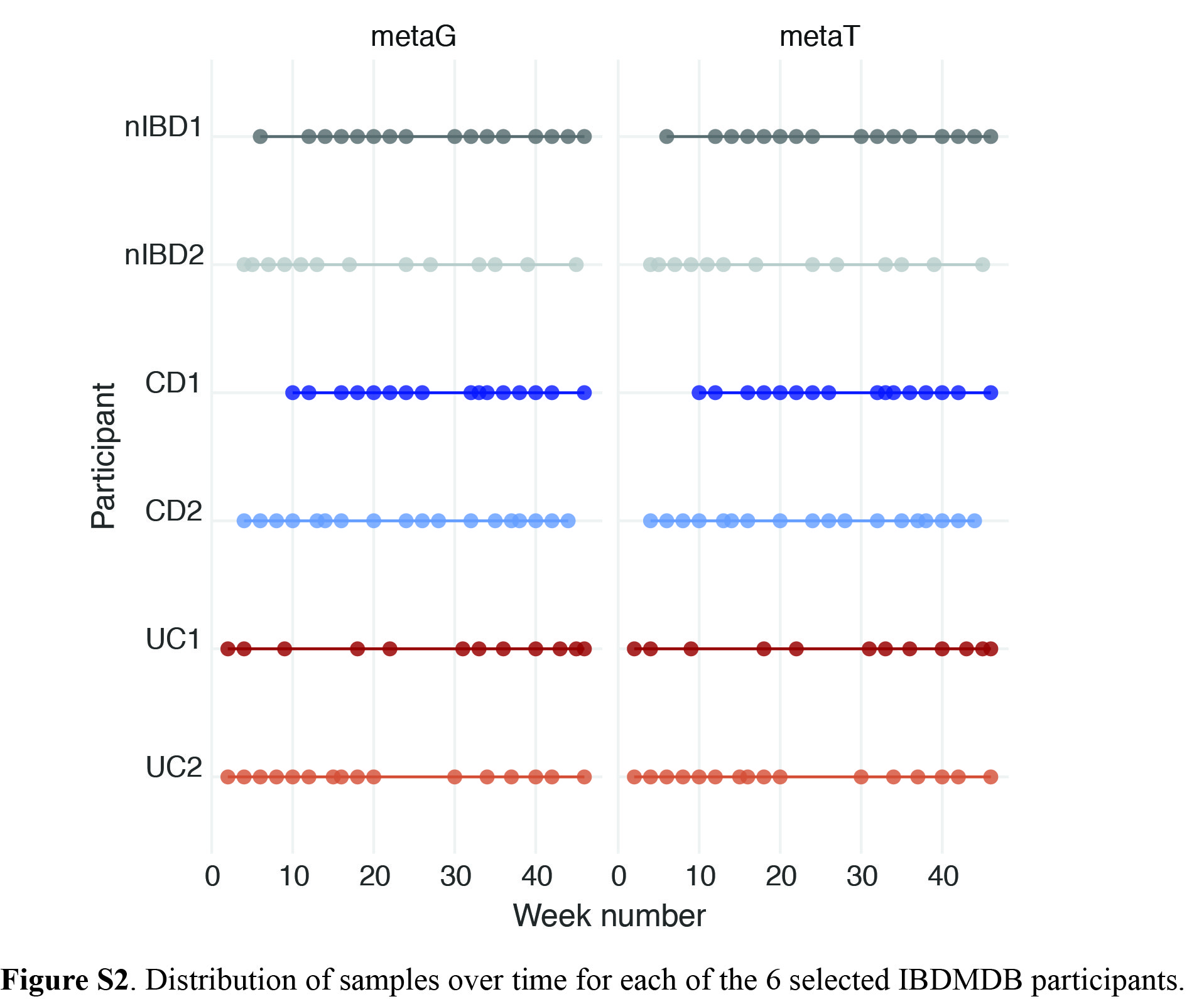

Supplement: Supplementary file 7 [file Image2.JPEG]

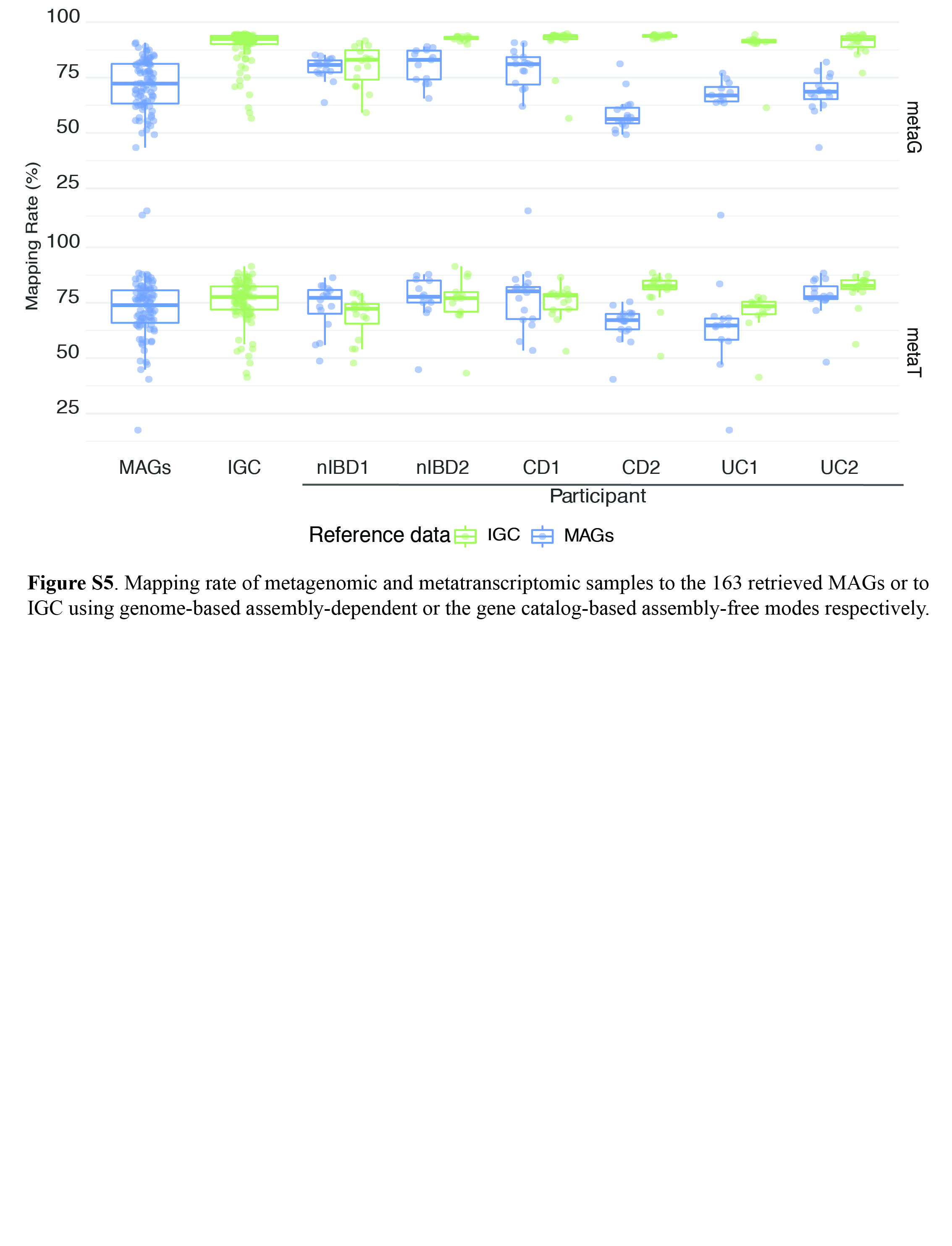

Supplement: Supplementary file 8 [file Image5.JPEG]

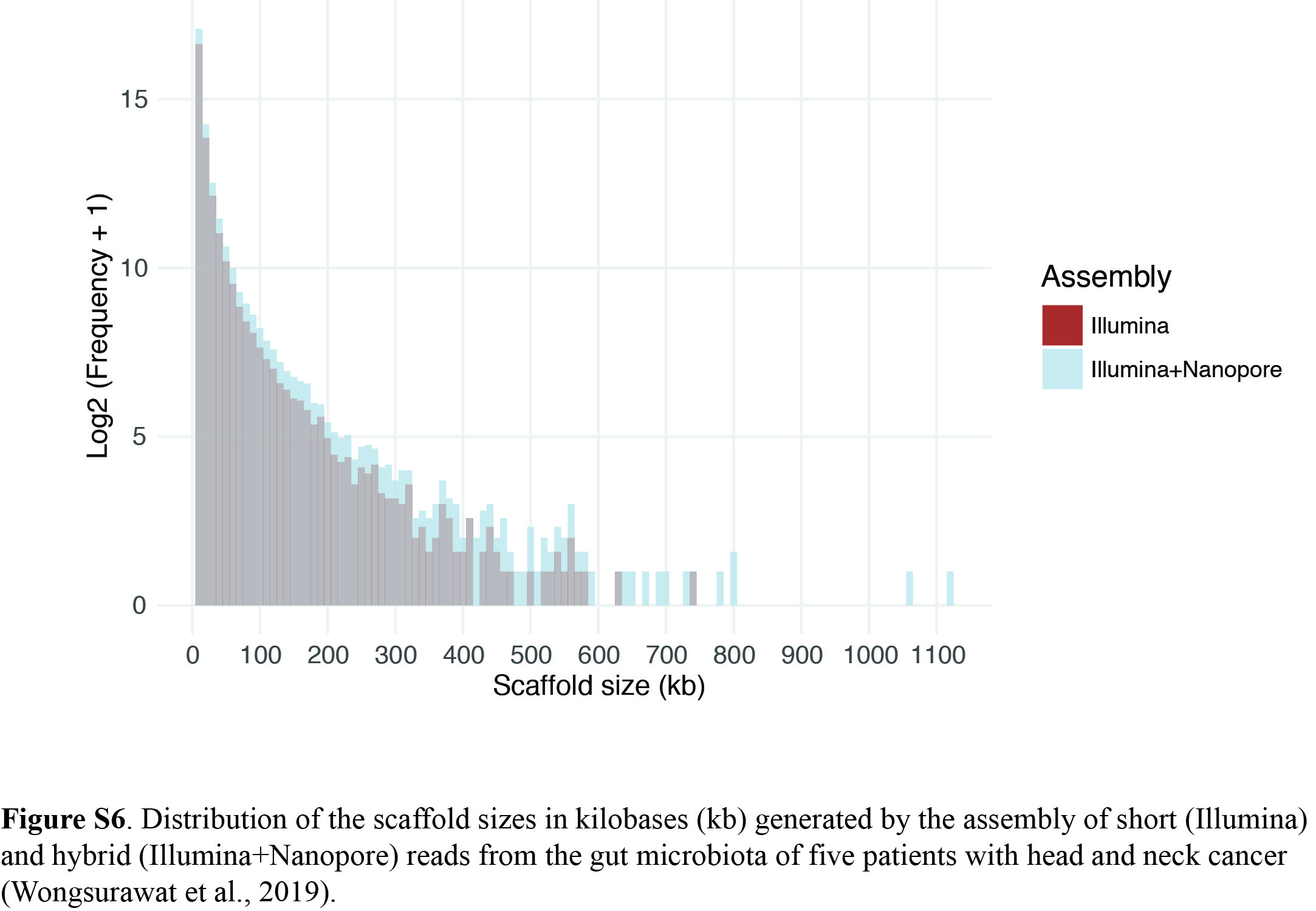

Supplement: Supplementary file 11 [file Image6.JPEG]
